# International Portuguese Nurse Leaders’ Insights for Multicultural Nursing

**DOI:** 10.3390/ijerph191912144

**Published:** 2022-09-25

**Authors:** Gisela Teixeira, Pedro Lucas, Filomena Gaspar

**Affiliations:** Nursing Research, Innovation and Development Centre of Lisbon (CIDNUR), Nursing School of Lisbon, 1600-190 Lisbon, Portugal

**Keywords:** cultural diversity, culturally congruent care, focus group, leadership, nursing, management, work environment

## Abstract

Cultural diversity among patients and healthcare workers in the Portuguese healthcare organizations will increasingly challenge nurse managers to develop favorable nursing work environments and to improve culturally congruent care. Aim: This study aimed to identify nurse managers’ interventions that improve favorable nursing work environments in multicultural nursing teams and culturally congruent care for patients, based on Portuguese nurse leaders’ experience in international settings. Methods: A qualitative and exploratory study was conducted as the first stage of a sequential exploratory mixed study design. A convenience sample of Portuguese nurses with leadership experience of multicultural teams was recruited to participate in one focus group. Qualitative data were recorded and transcribed for content analysis. Text segments were organized into themes and categories with the support of the qualitative software IRaMuTeQ. Results: Nurse managers’ interventions, such as adapting the leadership style, thanking nurses for their work, adjusting the unit to attend to patients’ worship practices, and supporting foreign nurses in learning the local language, were categorized into three main themes and five categories—transcultural nursing leadership assumptions, capitalizing nurses, improving culturally congruent care, team problems and strategies, and improving effective communication. Conclusions: These findings corroborate international studies, advocate for capable nurse managers to lead in a globalized world, and are suitable to develop a transcultural nursing leadership questionnaire.

## 1. Introduction

Portugal is a host country for more than half a million immigrants, so cultural diversity among patients and healthcare workers tends to be the rule rather than an exception in Portuguese healthcare organizations [1,2]. Access and healthcare service utilization of host societies are essential conditions for the health and well-being of these people, therefore, for their successful integration in the country. According to Oliveira & Gomes [3], immigrants face several barriers to access healthcare services, including discrimination, lack of information and low knowledge of the Portuguese healthcare system functioning, administrative barriers, cultural and linguistic barriers, and difficulties in the communication between them and the National Healthcare Service (SNS)’s employees and healthcare workers. These difficulties can demobilize immigrants’ demand for health services in Portugal or generate a decrease in the capacity of services to respond to their health needs, indirectly inducing inequalities in health protection.

The COVID-19 pandemic accentuated the inequalities in access to healthcare services that persist in European countries, which poses risks to societies in general [2]. Oliveira and Gomes [3] argue that countries must recognize that migratory flows induce necessary adjustments of healthcare workers and services to respond to the needs of cultural diversity, if they are to continue to improve health equity.

In the last few decades, Portugal has been, as well, a host country for foreign healthcare workers, mostly physicians and nurses, who have responded essentially to the shortage of healthcare human resources in the SNS and its asymmetric geographical distribution in Portugal. However, it should be viewed as well as a strategy to integrate cultural competencies in care delivery and to respond to the needs demanded by cultural diversity [3]. According to the Portuguese Board of Nursing statistical data for the year 2021 [4], about 2.5% of board members were foreign nurses working in Portugal.

Cultural diversity among patients and healthcare workers in Portuguese healthcare organizations will increasingly challenge nurse managers to develop favorable nursing work environments for multicultural nursing teams that are conducive to the delivery of culturally congruent care to patients from different cultural backgrounds, in order to enhance professional satisfaction and retention, and to improve health outcomes. The nursing work environment is understood to be the set of characteristics of the work environment that facilitate or restrict nurses’ professional practice [5]. Culturally congruent care is the use of sensitive, creative and meaningful care practices that match patients’ values, beliefs and lifestyles for beneficial and satisfying healthcare or to assist them in difficult life situations, disability or death [6].

Favorable nursing work environments are essential to improve nurses’ satisfaction and retention; to decrease their burnout in organizations; to improve the quality of care, patients safety, the experience of care in hospital settings, patients’ satisfaction with nurses’ communication; to reduce mortality and decrease costs in organizations; and to increase their rating and recommendation index [7,8,9,10]. The international literature highlights nurse managers’ interventions in multicultural nursing work environments at the organizational, unit and nurses’ level towards patients’ care and towards team’s relationships [11].

This study aimed to identify nurse managers’ interventions that improve favorable nursing work environments to multicultural nursing teams and culturally congruent care to patients, based on Portuguese nurse leaders’ experience in foreign countries. It is part of a Ph.D. research project in nursing leadership, which aims, among other aims, to develop, validate and apply a questionnaire that measures nurse managers’ leadership behaviors in multicultural nursing work environments in Portugal.

## 2. Materials and Methods

A sequential exploratory mixed study was designed to explore a reality poorly studied [12] in the field of Portuguese nursing leadership and management. Studies with this design start with the collection and analysis of qualitative data, followed by the collection of quantitative data. This strategy is often selected to develop a rigorous quantitative instrument, wherein the findings from a qualitative study can be used to design a quantitative study, provide preliminary information about its psychometric properties and prepare it for future use in other samples for poorly investigated topics [13].

A qualitative and exploratory study was conducted to identify nurse managers’ interventions that develop favorable nursing work environments for multicultural nursing teams and to improve culturally congruent care to patients, guided by the research question: *What nurse managers’ interventions contribute to develop favorable nursing work environments to multicultural teams and to improve culturally congruent care to patients from different cultural backgrounds?* Its findings will be used to formulate items for a Portuguese transcultural nursing leadership questionnaire, for further validation in the quantitative stage of the study by the means of an exploratory factor analysis.

### 2.1. Sampling

For sampling purposes, Portuguese nurse managers, team leaders and specialist nurses with at least one year of experience leading multicultural nursing teams were considered. Following the recommendation of five to 10 participants for focus groups [14], a convenience sample of nine Portuguese nurses that attended these criteria was recruited [13]. These nurses received an email with an explanation of the study and invitation to participate. Those who expressed interest to participate in the study were clarified when doubts arose.

A total of five Portuguese nurses with leadership experience in Saudi Arabia (*n* = 3), United Arab Emirates (*n* = 1) and the United Kingdom (*n* = 1) signed a consent form to voluntarily participate in the study. Three nurses were female, and the mean age of the group was 37.6 years (SD = 2.2). One participant had a Ph.D. degree; three had a Master’s degree, and one a Bachelor’s degree. The group had a mean of 4 years (SD = 2.3) of experience leading multicultural nursing teams, three participants as head nurses, one participant as a director of one hospital unit and one participant as a specialist nurse.

### 2.2. Focus Group

A focus group is a technique to collect qualitative data by the mean of a focused discussion with a group of people who share common characteristics related to the topic under study [14], which allows the researcher to understand how the participants talk about the phenomenon of interest [15]. It was understood that this methodological option would stimulate the sharing of experiences among participants and, consequently, improve the richness of the data.

A semi-structured interview script was developed with questions designed to capture participants’ perceptions about transcultural nursing leadership, nurse managers’ interventions to develop favorable nursing work environments for multicultural nursing teams, and interventions to improve culturally congruent care to patients from different cultural backgrounds. The interview was performed online with Microsoft Teams^®^ since participants were located in different countries and recorded with all participants’ consent.

### 2.3. Ethical Considerations

This study was approved by the Ethics Committee number 216/2022/CE. In order to safeguard the fundamental ethical principles that ensure respect for the participants’ dignity and autonomy [13], all participants’ questions were clarified, their participation in the study was voluntary, and a consent form was signed. The anonymity of participants and data confidentiality were guaranteed.

### 2.4. Data Analysis

The interview was transcribed and sent to the participants. No amendments were made by them. The transcript constituted the *corpus* analysis for content analysis [16]. According to the order of their first intervention in the focus group, participants’ speeches were identified in the transcript as P1, P2, P3, P4 or P5.

Once prepared, several readings of the transcribed interview were made to understand the content and identify possible meaning units. Subsequently, the coding phase was performed. The software Interface de R pour les Analyses Multidimensionnelles de Textes et de Questionnaires (IRaMuTeQ) was used to support this phase and strengthen the robustness of data analysis.

IRaMuTeQ has been used to process qualitative data, namely in the stage of coding and organizing the data in content analysis [17]. To process the *corpus* analysis in the software, it was transformed into a monothematic text *corpus* according to required rules [18]. Hierarchical descent classification (HDC) was selected as the method for data processing and to organize it into themes and categories. The feasibility of the HDC was assessed by the percentage of text segments retained, considering a minimum of 70% acceptable [19]. The statistical significance of the words in the retained text segments and its Chi-square (χ^2^) equal or greater than five were also evaluated, value of reference that represents a good delimitation between the categories [19].

The classification of the themes and categories resulted from the interpretation of the set of statistically significant words identified in each category. IRaMuTeQ allows the visualization of the text segments in which each of these words is inserted, thus facilitating their location in the *corpus* analysis, the identification of the context units and verifying which situation these words were used by the participants and what meanings were assigned to them. Text segment interpretation in each category resulted in its breakdown into subcategories.

Findings were analyzed in light of the evidence on management and leadership in multicultural nursing work environments.

## 3. Results

The focus group interview was conducted in March 2022 and lasted two hours. All participants contributed with their experience; they were active and respected others’ experience and opinions.

**Figure 1:**
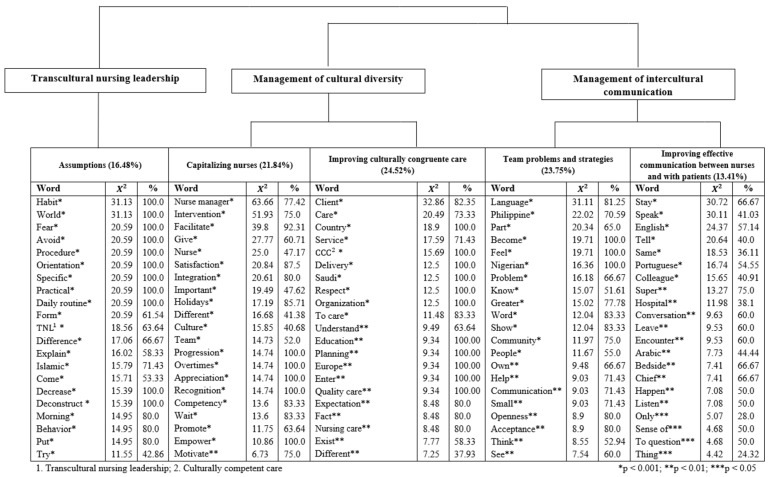
Perceptions of Portuguese nurse leaders about nurse managers’ interventions regarding favorable nursing work environments for multicultural nursing teams and regarding culturally congruent care to patients from different cultural backgrounds: themes, categories and statistically significant words.

The HDC method retained 79.33% of the corpus’ text segments to build three main themes and five categories, named according to the contextualization and interpretation of their statistically significant words (Figure 1).

A total of 31 subcategories were identified from the analysis of the text segments of each category.

### 3.1. Assumptions (of Transcultural Nursing Leadership)

Six assumptions of transcultural nursing leadership (Table 1) were identified. According to participants, transcultural nursing leadership requires nurse managers who are able to adapt and adjust their management practices according to the expectations of the people led; are culturally humble and knowledgeable of nurses’ cultural backgrounds and how it influences their care practices and behaviors; are able to bring people together, impartial in their decision-making and capable of guiding a multicultural team to achieve common goals. It was also pointed out that there are differences in nursing practice between nurses from different countries that should be minimized by the nurse manager, based on strategies to standardize it, such as using international protocols, monitoring nurses’ compliance with quality and safety standards, keeping the team’s skills updated and creating orientation programs to develop specific clinical skills.

### 3.2. Capitalizing Nurses

This category encompasses participants’ perceptions of nurse managers’ measures that increase satisfaction, facilitate integration and promote the valorization and retention of migrant nurses (Table 2).

Planning nurses’ leave and schedules according to their family and religious priorities, extra income, equal opportunity for training and progression, and impartiality in decision-making are conditions that seem to satisfy migrant nurses. From participants’ perspective, nurse managers facilitate the integration of migrant nurses if they show interest and understanding of their culture; know and use words in their languages; listen, receive and accompany them; manage their expectations; create integration programs; assign a preceptor of the same nationality; extend the integration period if necessary; and develop social activities.

Thanking daily, empowering, providing opportunities to develop skills and involving migrant nurses in projects are ways to value and recognize their skills, with a positive impact on retention.

### 3.3. Improving Culturally Congruent Care

This category captures participants’ perceptions of the conditions that a nurse manager should ensure to promote outstanding care to patients from different cultural backgrounds, which includes providing cultural training to nurses and encouraging the development of their cultural sensitivity, planning and organizing care according to patient’s culture, managing expectations, adapting food and cleaning services to patients’ needs, ensuring resources and structures for their religious and linguistic needs and involving relatives in patient care (Table 3).

### 3.4. Team Problems and Strategies

This category identifies the main problems in nursing work environments resulting from communication differences between nurses coming from different countries and nurse managers’ strategies to prevent or solve them (Table 4). According to participants, in multicultural work environments, there is a propensity for nationalities’ segregation and communication between health professionals in their mother tongues, triggering situations of bullying and discrimination towards other nationalities. Non-verbal communication can also make it difficult to interpret messages transmitted between different nationalities. A need was stressed for the nurse manager to identify these situations early, prohibit the mother tongue if different from the local, assign representatives of different cultures to be cultural mediators, build teams with mixed nationalities and encourage cultural mediation programs within the organizations to assist the management of these problems. If discriminatory behaviors are recurrent, they should be weighed in the performance appraisals of those practicing them. Nurse managers should also develop communication skills to improve their understanding of others nationalities’ verbal and non-verbal language and should demonstrate examples of respect to their teams.

### 3.5. Improving Effective Communication between Nurses and with Patients

This category contextualizes nurse managers’ interventions that improve effective communication between healthcare workers and between these and patients from different nationalities (Table 5). According to participants, to promote a professional communication between nurses that speak different languages, the nurse manager must always communicate in the official language of the organization; negotiate the use of the mother tongue outside the clinical area; and, instead of the integration being performed by a colleague of the same nationality, it must be assigned a preceptor of a different nationality to force communication in the language recommended by the organization. In order to improve the positive communication between migrant healthcare workers and their patients, the need to learn the local language was underlined by, for instance, nurse managers, who should encourage and support staff participation in a language course.

## 4. Discussion

Several nurse managers’ interventions were identified related to transcultural nursing leadership assumptions, capitalizing nurses, culturally congruent care improvement, team problems and strategies and the enhancement of effective communication. Leininger’s theory of culture care, diversity and universality is a theoretical framework suitable to understand the cultural diversity in nursing practice work environments. It supports research to uncover administrative patterns, assists formal leaders in problem-solving and decision-making, identifies factors that influence the recruitment and retention of foreign nurses, manage cultural pain, and plan interventions that promote care aligned with patients’ expectations [20]. Leininger [21] defines transcultural nursing administration as a creative and intelligent process to assess, plan, make decisions and formulate policies that facilitate education and the delivery of clinical services congruent with the values, beliefs and lifestyles of people from different or similar cultures, which sustains our findings.

The perceptions of the study’s participants regarding transcultural nursing leadership are in line with Leininger’s definition, as well as with the cross-cultural leadership definition as a process that influences a global community to adopt a shared vision and work towards a common goal [22], which requires aptitude to adapt to the complexity of other cultures and the capability to understand them, accept them and respond effectively to their differences [23].

It is important for nurse managers to develop cultural knowledge to facilitate the assessment and understanding of nurses’ behaviors and care practices and, consequently, to plan and make decisions that promote closeness between people and the delivery of quality care to patients. Cultural knowledge and cultural humility, as a spiritual outcome of becoming culturally competent [24], will provide the nurse manager with a greater ability to deconstruct differences, considering that, in order to build bridges across cultural boundaries, this ability to perceive, analyze and decode behaviors and situations in multiple cultural contexts and to understand the dynamics of the interactions between different cultures is essential [25]. Therefore, according to Vilas-Boas and Davel [26], interculturality in organizations should guide the way practices are built and the way leaders decide, act and interact; thus employees’ culture is a variable that managers should always consider in their leadership practices.

The differences in nursing practice among foreign nurses was a concern highlighted in the focus group and nurse manager’s strategies to standardize it were discussed. Expectations for practice and standards of safety and quality of care may be interpreted differently across cultures [27]. Therefore, several strategies are needed to guide immigrant nurses in the country’s culture before starting their clinical practice [28], namely specific orientation programs, as pointed out by one of the participants of our study. According to other studies, these programs decrease the differences in practice between the nurse’s country of origin and the host country [27], minimize the impact of the challenges and difficulties experienced, improve well-being and quality of care and mitigate the risks to patients’ safety [29].

Our findings regarding nurse managers’ interventions support other studies that conclude the factors that most affect immigrant nurses’ job satisfaction levels are the nurse managers’ lack of response to their cultural needs [30], the salary [31,32], the opportunities for training and progression [31,33,34] and the nurse managers’ favoring practices [35]. Knowing and being sensitive to nurses’ needs and planning shifts considering their religious and cultural events, as stated in the focus group, meet the recommendations for multicultural healthcare organizations that benefit the well-being of their migrant workers [36]. Nationality-based favoritism influences negatively nurses’ satisfaction [35] and contributes to discrimination in healthcare organizations [37,38,39].

The difficulty of progression to management and leadership positions has emerged, associated with favoritism, discrimination and racism [37,38,39,40], particularly when managers’ support for professional development is not provided in a similar way to all nurses. Prejudices and stereotypes of a nurse manager may lead to biased decisions-making, so an awareness of their own beliefs and how they influence their management practices is necessary [41]. Thus, impartiality is a core characteristic of transcultural nursing leadership that was identified in our study as an important assumption.

It is desirable that nurse managers support the development of a non-discriminatory environment to improve the quick integration of immigrant nurses [42]. According to Rovito [27], the way immigrant nurses perform their role according to the expected norms and expectations depends on how they cope with the cross-cultural challenges they experience. To ensure their successful transition to the host country, it is necessary to know the culture of care practice in their origin countries and plan strategies in advance to resolve difficulties during their integration [43]. Clinical mentoring programs, assigning preceptors of the same nationality during the probation period and extending it if necessary are strategies for these nurses identified in our study, confirmed by others [36,44]. Planning of social activities, as mentioned in the focus group, enhance harmony, cooperation, empathy, understanding of differences and respect, diminishing potential prejudice and stereotypes, favoring a more inclusive environment [45,46], promoting satisfaction and facilitating the integration of migrant nurses [31,47].

Saying “thank you” is believed to be a way to value the work performed with medium- to long-term benefits. Evidence shows that feeling valued motivates immigrant nurses, helps them to cope with stress factors [39] and nurtures feelings of achievement [48]. When nurses feel well-received and supported, they tend to stay in the hospital that recruited them as a sign of gratitude and loyalty [49], associated with the affective bond mentioned by one of our study’s participants. Valuing immigrant nurses through gratitude and recognizing their potential and giving them opportunities for training and progression as verbalized in the focus group may be catalysts for better retention rates in the long term.

The role of nurse manager in improving some essential conditions in the delivery of outstanding care to patients of different nationalities and religions, such as training in culture, was highlighted in our study. Providing trainings about culture helps nurses to know the prevalence of diseases in different geographical regions and cultural contexts, to improve their understanding of some health conditions and to plan and deliver care accordingly [50]. The planning and organization of care adjusted to patients’ culture imply two central conditions: the assessment of patients’ cultural needs and a broad cultural knowledge that eases culturally appropriate decision-making and responds to the identified needs in due time. Consistent with El Amouri and O’Neill [51] and Chae and Park [52], cultural assessment systems and tools are required to facilitate the identification of the needs and the planning of interventions that are culturally acceptable to patients.

Patients from different cultural backgrounds will have different expectations of care experience, and understanding these expectations can enable the planning and delivery of culturally and linguistically appropriate services to them [53]. The strategies identified in the focus group to address patients’ religious and linguistic needs meet the guidelines to implement culturally congruent nursing care in organizations [54] and the measures that improve the delivery of culturally and linguistically appropriate services [53]. Kitchen services that make meals appropriate to patients’ cultures and translation services are examples of organizational efforts that improve the quality of care [52] and support the perceptions and experiences shared in the focus group.

The inclusion of family as cultural mediators is also a strategy advocated to improve culturally sensitive and appropriate care practices in healthcare organizations [55]. Nurses need to understand the family’s care practices and how they can influence the care experience [56]. Embracing family in patient care benefits the patient, helps nurses to overcome communication difficulties, improves their cultural knowledge and strengthens their bond with the patient, strengthens the family’s trust in nurses and enables decision-making that promotes culturally congruent and ethical practices to patients [57].

Developing healthcare workers’ cultural sensitivity is also essential to ensure the quality and safety of care [58]. Cultural sensitivity requires valuing, respecting and admiring cultural diversity, and its improvement is central for healthcare workers to become culturally competent [59,60]. As a way to enhance this sensitivity, one of our participants recommended encouraging nurses to meet other cultural realities.

Understood as the intentional involvement in interactions with people from different cultural backgrounds [61], cultural encounters should be developed in the process of becoming culturally competent, which is essential to deliver effective and culturally sensitive services and to reduce disparities in the quality of care and health of different populations [62]. Becoming culturally competent also has positive consequences for care providers. According to Sharifi et al. [60]’s findings, a continuous cultural encounter allows nurses to acquire greater knowledge and awareness of different cultures, greater cultural skill, improve the culturally congruent care they provide and gain patients’ trust and respect. It was also highlighted that nurse managers have a key role in preventing and solving problems related to discrimination and improving effective professional communication between healthcare workers and with patients. Mosed [63] argues that communication in healthcare organizations is always related to patient care and any failure due to misunderstanding of culture can lead to poor health outcomes. In nursing work environments where verbal and nonverbal communication may vary significantly, it is critical that nurse managers invest in developing intercultural communication skills, understand the implied messages in others’ body language and speech [64], be aware of their own and others’ communication styles and consider ways to adapt [65,66].

Nurses from ethnic minorities need to feel supported and be assured that incidents related to racism, discrimination, physical or verbal abuse and harassing behaviors in the workplace will be investigated and that effective measures will be instituted to decrease the risk of recurrence [33,36,37,67]. It is essential to create opportunities for nurses to analyze how their biases influence attitudes and behaviors during cross-cultural encounters [68], and performance appraisal, as identified in our study, may be an appropriate opportunity.

Encouraging nurses to communicate in the official language recommended by the organization is a strategy identified in our study that, according to Burner [45], eliminates the possibility of other colleagues feeling uncomfortable or excluded in the work environment. A policy that monitors communication in the workplace, such as the English language mentioned by the participants, can be a measure that ensures the inclusion of all team members [69]. From our point of view, it can also standardize communication during clinical practice and prevent the miscommunication that threatens safety and continuity of care.

Different languages, accents, speech speeds and communication styles are barriers that affect not only the cooperation between nurses but also the nurse–patient relationship [39]. Differences in language, culture and religion make the communication of this dyad difficult, negatively influencing their relationship, adversely affecting patient’s safety and satisfaction and making patient-centered care unviable [70,71]. Encouraging foreign nurses and facilitate their attendance in language courses, as mentioned in the focus group, and encouraging autochthonous colleagues to help them adapt their communication to the host country are recommendations to be implemented in services wherein multicultural healthcare teams work [36]. Learning the Portuguese language is considered an important dimension for immigrants’ integration in Portugal. Although not mandatory, the country has several programs and resources in this field [2].

## 5. Limitations

Four of five participants had leadership experience in Middle Eastern countries, where cultural and religious contexts are different from those in Western countries. This must be taken into account when translating this study’s findings to the development of the leadership questionnaire to be applied in Portugal. We recommend to develop focus groups with Portuguese nurses in management positions in Western countries, with the purpose of exploring more interventions that improve favorable nursing work environments and the delivery of culturally congruent care in multicultural healthcare settings, and to compare its findings with the ones from this study.

## 6. Implications

Rosa and Shaw [72] advocate for the ideas that we all share a common humanity and that nurses’ practice must be competent in caring of populations from different nations and continents, regardless of they are formal leaders or not. Our findings appeal for a greater consciousness that we do live in a globalized world and that more research must be done in order to deepen and disseminate the knowledge in this field, such as the organizational behaviors, job satisfaction and retention of multicultural nursing teams and its impact on patients’ outcomes in Portugal. It appeals as well to educating and training future nurse leaders and managers under a transcultural point of view in Portuguese universities.

## 7. Conclusions

The aim of this study was to identify nurse managers’ interventions that improve favorable nursing work environments in multicultural nursing teams and culturally congruent care for patients from different cultural backgrounds. Several nurse managers’ interventions regarding transcultural nursing leadership, the management of nurses’ and patients’ cultural diversity and the management of intercultural communication were identified, suitable for the formulation of a nursing leadership questionnaire’s items.

Due to the increased cultural diversity in Portuguese healthcare organizations, our findings can also contribute to building guidelines for nurse leaders and managers in Portugal and to develop projects aiming to improve the quality of nursing work environments and culturally congruent care in multicultural healthcare settings.

Our findings corroborate international studies and advocate for the idea that capable nurse managers should lead in a globalized world.

## Figures and Tables

**Table 1:** Subcategories of the “Assumptions of transcultural nursing leadership” category and participants’ quotes.

Category	Subcategories	Quotes
Assumptions of transcultural nursing leadership	Adaptation	“It has to be a leadership that is able to adapt” (P1). “Transcultural leadership is the challenge and adaptation of different cultures caring for different cultures” (P2).
	Cultural knowledge and humility	“Leading a multicultural team requires a sensitivity and humility to understand the needs that people have, not only linked to culture, religion, but to habits, principles” (P3). “Recognizing the differences of each culture and realizing the importance of the culture of each nurse and each team member on a daily basis” (P1).
	Deconstruction of differences	“We are all equal in the world and I think it is important for those who come from such different places to realize that although people are extraordinarily different, with extraordinarily different habits, I try to deconstruct, break down all this difference” (P4).
	Impartiality	“A leadership that is transcultural has to avoid unconscious bias” (P1). “Here we cannot give an opportunity to have more overtimes otherwise we can be accused of discrimination and favoritism” (P2).
	Orientation for goals	“Conducting them in a harmonious way to achieve the end, our goal, which is such outstanding care” (P5).
	Standardizing nursing practice	“The standardization of a nursing procedure, despite being exactly the same all over the world, the way it is performed is different” (P4). “Those who do not have a specific pediatric cardiology ICU experience, we have created a program […] to try to compensate a little bit that difference” (P3).

**Table 2:** Subcategories of the “Capitalizing nurses” category and participants’ quotes.

Category	Subcategories	Quotes
Capitalizing nurses	Professional satisfaction	“Give the priorities for holidays at the most important times, mainly from a religious point of view […] we quickly come to conclusion when we get close to these teams that what the staff values the most is the holidays and the schedules” (P3). “We can give a little bit here to those who want more money […] give an opportunity to have more overtimes” (P5). “To give our nurses the option of having a similar level of training, similar opportunities, it’s not just one nationality that stays as charge nurse” (P2).
	Integration	“An intervention that is important for us, for transcultural leaders, is to understand the expectations of our health professionals” (P2). “If the probation period is required to take longer, it will take longer” (P4). “Choosing someone of the same nationality also to help to integrate and help to overcome some problems in the beginning” (P5). “Whenever there are these kinds of farewell parties, it is a little bit a moment of sharing experiences and culture and I think that this has worked very well and people get to know, end up respecting each other a little more and are able to anticipate the others’ needs” (P3).
	Appreciating and recognizing	“I go to all my team members and say thank you for your work today, thank you to work so hard, and that makes the difference, because they feel recognized and they feel valued” (P1). “[…] they wanted to stay in my hospital […] I empowered them and that’s what they knew they were going to develop, their skills that they wouldn’t get in other places” (P2).
	Retention	“[…] they have a project in common with the unit, they will feel that they have a mission and I think they feel better and will not want to leave the unit so easily” (P3). “Maybe those charge nurses didn’t leave because they felt an affective bond” (P4). “Most foreign nurses love training, they want to do a master’s degree, they want to do everything they can (…) it’s career progression, which is sometimes complicated when you’re a foreign nurse here (…) being a leader open to all cultures. For me, these are the three pillars: education, compassion and progression.” (P1)

**Table 3:** Subcategories of the “Improving culturally congruent care” category and participants’ quotes.

Category	Subcategories	Quotes
Improving culturally congruent care	In-services about culture	“An education program in the unit that promotes multicultural nursing care, encourages for example, the article of the week related to culture X, Y or Z” (P1).
	Cultural sensitivity development	“Encourage staff to meet new cultures […] to widen the boundaries, to know other things, this will make it much easier for them to adapt to what is different” (P4).
	Care planning and organization according to patient’s culture	“When I have teenagers, I have to have that notion, we cannot assign male nurses to care of girls [Muslim]” (P3).
	Management of expectations	“… one of the important points in relation to quality is this scope of service has to be accessible to the patient, understood by the patient, it’s one of the things we do on admission, and so both parties know the expectations, what is expected from the nurse and what the patient can expect from the team itself” (P2).
	Resources for religious and linguistic needs	“Adjust the unit or adjust the department, the hospital, to have places to exercise their worship” (P5). “Translators for patient, in case the patient doesn’t know how to speak our language” (P2).
	Adaptation of auxiliary services	“Meal times during festive seasons or, for example, during Ramadan, have to be adjusted” (P2).
	Family inclusion	“When we include the family in the care (…) it makes things much easier for us” (P1).

**Table 4:** Subcategories of the “Team problems and strategies” category and participants’ quotes.

Category	Subcategories	Quotes
Team problems and strategies	Discrimination	“Using one’s own language gives rise to this kind of discrimination, which ends up causing this kind of discrimination and even some kind of bullying between nationalities” (P3).
	Prejudice	“I have a nurse assistant who doesn’t get on with them [Nigerian nurses], and I’m trying to demonstrate that it’s a conditional bias, that she’s only doing that to them and not to the rest of the team” (P1).
	Speech misunderstanding	“People also did not understand me the same way they understand me in Portugal (…) We all have the same culture and there are things that are obvious and are quickly understood. And when we get to a multicultural team, we also have that barrier” (P3).
	Early identification	“The early identification of those situations, i.e., a good observation and identification skills. Awareness also, i.e., being alert for that kind of problems, anticipation” (P2).
	Culture mediation programs	“There is an RCN program called Cultural Ambassador (…) when there are problems they will investigate, they will understand if there is a cultural reason for that problem to have existed. So, as leaders, encouraging these kinds of programs is essential, because it will help us to understand when there is a problem, how we can act” (P1).
	Prohibition of the mother tongue	“These problems that arise between different cultures come mainly from the language and that’s why we don’t allow them to speak their own language” (P3).
	Performance appraisal	“We do a performance appraisal every year and if there is this kind of behavior, it has to be talked about” (P1).
	Mixed working teams	“(…) I am always very careful to make the teams to bring all the nationalities together so they work together, they have the same goal, they work together and I think that helps a lot” (P3).
	Nurse manager communication skills	“I myself had to develop my communication skills to adapt to them” (P2).
	Nurse manager as a role model	“You have to give the example and they will follow the leaders” (P2).

**Table 5:** Subcategories of the “Promoting effective communication between nurses and with patients” category and participants’ quotes.

Category	Subcategories	Quotes
Promoting effective communication between nurses and with patients	Being a model for communication	“We had, as nurse managers, to be an example, so between us, we always spoke in English” (P2).
	Negotiate the mother tongue utilization	“(…) among friends they can speak their language. In the clinic they don’t speak, they have to speak the official language, it’s English and it’s a language that everybody understands” (P3).
	Assign a preceptor of a different nationality	“I try not to put a new staff to be integrated by a staff of the same nationality (…) because if they are with someone of the same nationality, they will end up speaking the same language and they will not get used to speak in English” (P3)
	Support the learning of the local language	“Motivate to take that course [of Arabic language] and make it possible to go (…) language is a linguistic barrier, it’s very distressing and I think it’s important to be able to communicate correctly with the patient to have that delivery of outstanding care” (P3).

## Data Availability

The data presented in this study are available on request from the first corresponding author.

## References

[1] Lopes J.C., Lopes J., Santos M., Matos M., Ribeiro O. (2009). Organizações de Saúde Multiculturais. Multiculturalidade: Perspectivas da Enfermagem Contributos Para Melhor Cuidar.

[2] Oliveira C. (2021). Indicadores de Integração de Imigrantes: Relatório Estatístico Anual 2021.

[3] Oliveira C., Gomes N. (2018). Migrações e Saúde em Números: O Caso Português, Caderno Estatístico Temático #2.

[4] Anuário Estatístico 2021. https://www.ordemenfermeiros.pt/estatística-de-enfermeiros/.

[5] Lake E. (2002). Development of the practice environment scale of the Nursing Work Index. Res Nurs Health.

[6] Leininger M., Leininger M., McFarland M. (2002). Essential Transcultural Nursing Care Concepts, Principles, Examples, and Policy Statements. Transcultural Nursing: Concepts, Theories, Research, and Practice.

[7] Lucas P., Jesus E., Almeida S., Araújo B. (2021). Validation of the Psychometric Properties of the Practice Environment Scale of Nursing Work Index in Primary Health Care in Portugal. Int. J. Environ. Res. Public Health.

[8] Kutney-Lee A., Wu E., Sloane D., Aiken L. (2013). Changes in hospital nurse work environments and nurse job outcomes: An analysis of panel data. Int. J. Nurs. Stud..

[9] You L., Aiken L., Sloane D., Liu K., He G., Hu Y., Jiang X., Li X., Liu H., Shang S. (2013). Hospital nursing, care quality, and patient satisfaction: Cross-sectional surveys of nurses and patients in hospitals in China and Europe. Int. J. Nurs. Stud..

[10] Lake E., Sanders J., Duan R., Riman K., Schoenauer K., Chen Y. (2019). A Meta-Analysis of the Associations Between the Nurse Work Environment in Hospitals and 4 Sets of Outcomes. Med. Care.

[11] Teixeira G., Gaspar F., Lucas P. (2022). Nurse manager’s role in promoting culturally competent work environments in nursing: An integrative review. New Trends Qual. Res..

[12] Creswell J.W., Creswell J.D. (2018). Research Design: Qualitative, Quantitative, and Mixed Methods Approaches.

[13] Grove S., Gray J. (2019). Understanding Nursing Research. Building an Evidence-Based Practice.

[14] Krueger R.A., Casey M.A. (2015). Focus Groups: A Practical Guide for Applied Research.

[15] Silva I.S., Veloso A.L., Keating J.B. (2014). Focus group: Considerações teóricas e metodológicas. Rev. Lusófona Educ..

[16] Bardin L. (2016). Análise de Conteúdo.

[17] Klamt L., Santos V. (2021). The use of the IRAMUTEQ software in content analysis—a comparative study between the ProfEPT course completion works and the program references. Res. Soc. Dev..

[18] Tutorial Para Uso Do Software Iramuteq (Interface De R Pour Les Analyses Multidimensionnelles De Textes Et De Questionnaires). http://iramuteq.org/documentation/fichiers/tutoriel-portugais-22-11-2018.

[19] Mendes A.M., Tonin F.S., Buzzi M.F., Pontarolo R., Fernandez-Llimos F. (2019). Mapping pharmacy journals: A lexicographic analysis. Res. Soc. Adm. Pharm..

[20] Leininger M., McFarland M. (2006). Culture Care Diversity and Universality. A Worldwide Nursing Theory.

[21] Leininger M., Leininger M., McFarland M. (2002). Transcultural Nursing Administration and Consultation. Transcultural Nursing: Concepts, Theories, Research, and Practice.

[22] Hanges P.J., Aiken J.R., Park J., Su J. (2016). Cross-cultural leadership: Leading around the world. Curr. Opin. Psychol..

[23] Matveev A. (2017). Intercultural Competence in Organizations: A Guide for Leaders, Educators and Team Players.

[24] Abualhaija N. (2021). Clarifying cultural competence in nursing: A concept analysis approach. J. Cult. Divers..

[25] Cabrera A., Unruh G. (2012). Being Global: How To Think, Act, and Lead in a Transformed World.

[26] Vilas-Boas O., Davel E. (2018). Prática intercultural da liderança: Princípios e desafios da pesquisa empírica. Teor. E Prática Em. Adm..

[27] Rovito K., Kless A., Costantini S.D. (2022). Enhancing workforce diversity by supporting the transition of internationally educated nurses. Nurs. Manag..

[28] Safari K., McKenna L., Davis J. (2022). Transition experiences of internationally qualified health care professionals: A narrative scoping review. Int. J. Nurs. Stud..

[29] Ohr S.O., Holm D., Brazil S. (2016). The transition of overseas qualified nurses and midwives into the Australian healthcare workforce. Aust. J. Adv. Nurs..

[30] Taylor R. (1998). Check your cultural competence. Nurs. Manag..

[31] Itzhaki M., Ea E., Ehrenfeld M., Fitzpatrick J. (2013). Job satisfaction among immigrant nurses in Israel and the United States of America. Int. Nurs. Rev..

[32] Muhawish H., Salem O., Baker O. (2019). Job related stressors and job satisfaction among multicultural nursingworkforce. Middle East. J. Nurs..

[33] Mitchell J. (2009). Job Satisfaction and Burnout among Foreign-Trained Nurses in Saudi Arabia: A Mixed-Method Study. Ph.D. Thesis.

[34] Almalki M.J., Fitzgerald G., Clark M. (2012). Quality of work life among primary health care nurses in the Jazan region, Saudi Arabia: A cross-sectional study. Hum. Resour. Health.

[35] Saleh U., O’Connor T., Al-Subhi H., Alkattan R., Al-Harbi S., Patton D. (2018). The impact of nurse managers’ leadership styles on ward staff. Br. J. Nurs..

[36] Chen L., Xiao L., Han W., Meyer C., Müller A. (2020). Challenges and opportunities for the multicultural aged care workforce: A systematic review and meta-synthesis. J. Nurs. Manag..

[37] Likupe G. (2015). Experiences of African nurses and the perception of their managers in the NHS. J. Nurs. Manag..

[38] Ncube E. (2017). Influence of Leadership Styles on Expatriate Nurses’ Professional Integration in the UAE. Ph.D. Thesis.

[39] Schilgen B., Handtke O., Nienhaus A., Mösko M. (2019). Work-related barriers and resources of migrant and autochthonous homecare nurses in Germany: A qualitative comparative study. Appl. Nurs. Res..

[40] Henry L. (2007). Institutionalized disadvantage: Older Ghanaian nurses’ and midwives’ reflections on career progression and stagnation in the NHS. J. Clin. Nurs..

[41] Richard-Eaglin A. (2021). The Significance of Cultural Intelligence in Nurse Leadership. Nurse Lead.

[42] O’Callaghan C., Loukas P., Brady M., Perry A. (2018). Exploring the experiences of internationally and locally qualified nurses working in a culturally diverse environment. Aust. J. Adv. Nurs..

[43] Kelly S., Fowler C. (2019). Enhancing the recruitment and retention of overseas nurses from Kerala, India. Nurs. Stand..

[44] Munkejord M.C. (2019). Challenging the ethnic pyramid: Golden rules and organisational measures towards a more inclusive work environment. J. Nurs. Manag..

[45] Burner O.Y., Cunningham P., Hattar H.S. (1990). Managing a multicultural nurse staff in a multicultural environment. J. Nurs. Adm..

[46] Gillham D., De Bellis A., Xiao L., Willis E., Harrington A., Morey W., Jeffers L. (2018). Using research evidence to inform staff learning needs in cross-cultural communication in aged care homes. Nurse Educ. Today.

[47] Xiao L.D., Willis E., Jeffers L. (2014). Factors affecting the integration of immigrant nurses into the nursing workforce: A double hermeneutic study. Int. J. Nurs. Stud..

[48] Alshareef A.G., Wraith D., Dingle K., Mays J. (2020). Identifying the factors influencing Saudi Arabian nurses’ turnover. J. Nurs. Manag..

[49] Gullatte M.M., Jirasakhiran E.Q. (2005). Retention and recruitment: Reversing the order. Clin. J. Oncol. Nurs..

[50] McElroy J., Smith-Miller C., Madigan C., Li Y. (2016). Cultural awareness among nursing staff at an Academic Medical Center. J. Nurs. Adm..

[51] El Amouri S., O’Neill S. (2014). Leadership style and culturally competent care: Nurse leaders’ views of their practice in the multicultural care settings of the United Arab Emirates. Contemp. Nurse.

[52] Chae D., Park Y. (2019). Organisational cultural competence needed to care for foreign patients: A focus on nursing management. J. Nurs. Manag..

[53] Ogbolu Y., Scrandis D., Fitzpatrick G. (2018). Barriers and facilitators of care for diverse patients: Nurse leader perspectives and nurse manager implications. J. Nurs. Manag..

[54] Cho E., Sloane D., Kim E., Kim S., Choi M., Yoo I., Lee H., Aiken L. (2015). Effects of nurse staffing, work environments, and education on patient mortality: An observational study. Int. J. Nurs. Stud..

[55] Weerasinghe S., Maddalena V. (2016). Negotiation, Mediation and Communication between Cultures: End-of-Life Care for South Asian Immigrants in Canada from the Perspective of Family Caregivers. Soc. Work Public Health.

[56] Farias D., Almeida M., Gomes G., Lunardi V., Vieira E., Lourenção L. (2021). Family culture versus institutional hospital culture: A relation between two worlds. Rev. Esc. Enferm. USP.

[57] Murcia S., Lopez L. (2016). The experience of nurses in care for culturally diverse families: A qualitative meta-synthesis. Rev. Lat. Ame. Enf..

[58] Chang H., Yang Y., Kuo Y. (2013). Cultural sensitivity and related factors among community health nurses. J. Nurs. Res..

[59] Belintxon M., Carvajal A., Pumar-Méndez M.J., Rayon-Valpuesta E., Velasco T.R., Belintxon U., Dogra N., Vidaurreta M., Bermejo-Martins E., Lopez-Dicastillo O. (2021). A valid and reliable scale to assess cultural sensibility in nursing. Nurse Educ. Today.

[60] Sharifi N., Adib-Hajbaghery M., Najafi M. (2019). Cultural competence in nursing: A concept analysis. Int. J. Nurs. Stud..

[61] Campinha-Bacote J. (2002). The Process of Cultural Competence in the Delivery of Healthcare Services: A Model of Care. J. Transcult. Nurs..

[62] Chae D., Kim J., Kim S., Lee J., Park S. (2020). Effectiveness of cultural competence educational interventions on health professionals and patient outcomes: A systematic review. Japan J. Nurs. Sci..

[63] Mosed H., Periord M., Caboral-Stevens M. (2021). A concept analysis of intercultural communication. Nurs. Forum..

[64] Hunt B. (2007). Managing equality and cultural diversity in the health workforce. J. Clin. Nurs..

[65] Gill G.K., McNally M.J., Berman V. (2018). Effective diversity, equity, and inclusion practices. Healthc. Manag. Forum..

[66] Deardorff D.K. (2018). Exploring the Significance of Culture in Leadership. New Dir. Stud. Leadersh..

[67] Chambers C., Alexis O. (2004). Creating an inclusive environment for black and minority ethnic nurses. Brit. J. Nurs..

[68] Markey K., Prosen M., Martin E., Jamal H. (2021). Fostering an ethos of cultural humility development in nurturing inclusiveness and effective intercultural team working. J. Nurs. Manag..

[69] Ramji Z., Etowa J., St-Pierre I. (2018). Unpacking "two-way" workplace integration of internationally educated nurses. Aporia.

[70] Alshammari M., Duff J., Guilhermino M. (2019). Barriers to nurse-patient communication in Saudi Arabia: An integrative review. BMC Nurs..

[71] Sherwood G.D., Shaffer F.A. (2014). The role of internationally educated nurses in a quality, safe workforce. Nurs. Outlook.

[72] Rosa W., Shaw H., Rosa W. (2017). Global nurse citizenship: Toward a safe and inclusive civil society. A New Era in Global Health: Nursing and the United Nations 2030 Agenda for Sustainable Development.

